# The association between obstructive sleep apnea syndrome and sarcopenia: a meta-analysis

**DOI:** 10.3389/fmed.2026.1815694

**Published:** 2026-05-08

**Authors:** Yingxia Zhu, Yongli Luo, Runfen Du, Xiaoyan Dou, Minghui Yang, Wei Huang

**Affiliations:** 1 Department of Geriatrics, The Third People’s Hospital of Yunnan Province, Kunming, China; 2 Gucheng Township Health Center, Kunming, China; 3 Changshou Community Health Service Station in Panlong District, Kunming, China

**Keywords:** intermittent hypoxia, meta-analysis, obstructive sleep apnea syndrome, risk factor, sarcopenia

## Abstract

**Background:**

Obstructive sleep apnea syndrome (OSAS) and sarcopenia are prevalent conditions associated with aging and metabolic disorders. Emerging evidence suggests a potential link between intermittent hypoxia and skeletal muscle impairment, but findings remain inconsistent. We conducted a meta-analysis to clarify the association between OSAS and sarcopenia in adults.

**Methods:**

PubMed, Embase, Web of Science, Wanfang, and CNKI were searched for observational studies evaluating the association between OSAS and sarcopenia in adults. Odds ratios (ORs) with 95% confidence intervals (CIs) were pooled using random-effects models by incorporating the influence of potential heterogeneity. Subgroup and sensitivity analyses were performed to explore heterogeneity.

**Results:**

Eight cross-sectional studies comprising 13,331 participants (3,572 with OSAS) were included. Overall, OSAS was significantly associated with sarcopenia (OR 1.85, 95% CI 1.30–2.63; *I*^2^ = 62%). The association was stronger in Asian populations (OR 2.92) than in non-Asian populations (OR 1.33; *p* for subgroup difference < 0.001), and in older participants (mean age ≥ 64 years; OR 2.36 vs. 1.37; *p* for subgroup difference = 0.04). Studies using objective sleep assessment showed stronger associations than those using questionnaires or medical records (OR 2.78 vs. 1.33; *p* for subgroup difference < 0.001). Results were consistent in studies with NOS ≥ 8 (OR 1.82, 95% CI 1.23–2.70). Meta-regression analyses did not identify significant effect modifiers, although statistical power was limited by the small number of studies. No significant publication bias was detected (Egger’s *p* = 0.49).

**Conclusion:**

OSAS is associated with increased odds of sarcopenia in adults, particularly in older and Asian populations and in studies using objective sleep assessment. However, given that all included studies were cross-sectional and that diagnostic criteria for both OSAS and sarcopenia varied across studies, these findings should be interpreted cautiously and do not imply causality.

**Systematic review registration:**

https://www.crd.york.ac.uk/prospero/, identifier CRD420261324247.

## Introduction

Sarcopenia is a progressive skeletal muscle disorder characterized by age-related loss of muscle mass, strength, and physical performance (1, 2). It is now recognized as a distinct clinical entity and has been formally defined by several consensus groups, including the European Working Group on Sarcopenia in Older People (EWGSOP and its revised version EWGSOP2), the Asian Working Group for Sarcopenia (AWGS), and the Foundation for the National Institutes of Health (FNIH) Sarcopenia Project (2, 3). These definitions generally require documentation of low muscle strength (e.g., reduced handgrip strength), low muscle quantity or quality (e.g., decreased appendicular skeletal muscle mass index [ASMI] measured by dual-energy X-ray absorptiometry or bioelectrical impedance analysis), and in some criteria, impaired physical performance (e.g., slow gait speed) (4). Sarcopenia affects approximately 5–13% of community-dwelling older adults (5) and up to 30–50% of hospitalized or chronically ill populations (6), depending on diagnostic criteria and population characteristics. Importantly, sarcopenia is associated with increased risks of frailty, falls, disability, poor quality of life, hospitalization, and mortality (7). Identified risk factors include aging, physical inactivity, obesity, insulin resistance, chronic inflammation, hormonal alterations, and chronic diseases such as diabetes and chronic kidney disease, highlighting its multifactorial etiology (8).

Obstructive sleep apnea syndrome (OSAS) is a common sleep-related breathing disorder characterized by recurrent episodes of upper airway obstruction during sleep, resulting in intermittent hypoxia, sleep fragmentation, and sympathetic activation (9, 10). OSAS is typically diagnosed using polysomnography or validated sleep monitoring devices based on the apnea–hypopnea index (AHI) (11). It affects approximately 9–38% of the general adult population, with higher prevalence in older individuals and those with obesity (12). OSAS has been linked to adverse cardiovascular, metabolic, and neurocognitive outcomes, including hypertension, type 2 diabetes (T2D), coronary artery disease, and increased mortality (13). Emerging evidence suggests that chronic intermittent hypoxia, systemic inflammation, oxidative stress, hormonal dysregulation, and reduced physical activity associated with OSAS may contribute to skeletal muscle catabolism and functional decline, thereby providing biological plausibility for a link between OSAS and sarcopenia (14, 15). However, existing observational studies report inconsistent findings and are limited by heterogeneous diagnostic criteria and varying confounder adjustment (16–23). While prior syntheses have described the prevalence of sarcopenia among patients with OSAS (24), a quantitative synthesis evaluating the independent association between OSAS and sarcopenia using effect estimates remains lacking. Therefore, we conducted a meta-analysis to systematically evaluate the association between OSAS and sarcopenia in adults and to explore potential sources of heterogeneity across studies.

## Methods

The meta-analysis was carried out in accordance with established methodological guidance, following the principles outlined in the PRISMA 2020 statement (25) and the Cochrane Handbook for Systematic Reviews and Meta-Analyses (26), encompassing protocol planning, study selection, data extraction, statistical analysis, and reporting. The study protocol was registered prospectively in the PROSPERO database (registration number: CRD420261324247).

### Database search

We carried out a comprehensive literature search across PubMed, Embase, Web of Science, Wanfang, and China National Knowledge Infrastructure (CNKI) to identify eligible studies for inclusion. The search strategy was constructed using the combination of the following terms: (1) “sleep disordered breathing” OR “sleep breathing disorders” OR “sleep apnea syndrome” OR “obstructive sleep apnea” OR “obstructive sleep apnea syndrome” OR “obstructive sleep hypopnea syndrome” OR “OSAHS” OR “OSAS” OR “sleep apnea”; and (2) “sarcopenia” OR “muscle wasting” OR “muscle loss” OR “muscular atrophy” OR “muscle depletion” OR “sarcopaenia” OR “sarcopenic” OR “presarcopenia” OR “sarcopaenic” OR “lean body mass” OR “cross-sectional muscle area” OR “skeletal muscle depletion” OR “muscle mass” OR “muscle index.” Only full-text, peer-reviewed articles published in English or Chinese and conducted in human populations were considered eligible. We also manually examined the reference lists of relevant reviews and original studies to capture additional potentially eligible reports. Each database was searched from inception through January 24, 2026. The complete search strategies for all databases are provided in Supplementary File 1.

### Study inclusion and exclusion criteria

The selection of studies was guided by the PICOS principle:

P (Population): Adults aged ≥ 18 years from community-based or clinical settings were included without restriction by sex, ethnicity, or geographic region. Studies conducted in general populations as well as in specific disease populations (e.g., diabetes, chronic obstructive pulmonary disease, chronic kidney disease [CKD], or metabolic syndrome etc.) were eligible provided that adult data were separately extractable.
I (Exposure): The exposure of interest was OSAS, diagnosed according to the methods and criteria among the original studies, which included overnight polysomnography, validated sleep monitoring devices, risk scores, and self-reported diagnosis.
C (Comparator): The comparator consisted of adults without OSAS, as defined by the absence of diagnostic criteria for OSAS in the original studies.
O (Outcome): The outcome of interest was sarcopenia, defined according to established consensus criteria (e.g., EWGSOP, EWGSOP2, AWGS, or FNIH), which incorporate measures of low muscle strength (e.g., handgrip strength), low muscle mass (e.g., DXA-, BIA-, CT-, or MRI-derived indices such as SMI or ASMI), and/or impaired physical performance.
S (Study Design): Observational studies, including cross-sectional, case–control, prospective cohort, and retrospective cohort designs published in peer-reviewed journals, were included.

Studies were excluded if they: (1) included participants younger than 18 years; (2) involved central sleep apnea or mixed sleep apnea without separately reported data for OSAS; (3) did not clearly define OSAS or sarcopenia according to established diagnostic criteria; (4) assessed muscle mass alone without sufficient information to classify sarcopenia; (5) failed to report extractable effect estimates or provide adequate raw data for calculation; (6) were reviews, meta-analyses, editorials, letters, case reports, or case series; (7) were conducted in animals or experimental laboratory models; or (8) involved overlapping populations, in which case the study with the largest sample size was included.

### Study quality evaluation and data extraction

Two reviewers independently performed the literature search, screened eligible studies, assessed study quality, and extracted relevant data. Any disagreements were resolved through discussion, and when necessary, by consulting the corresponding author. Study quality was appraised using the Newcastle–Ottawa Scale (NOS) (27). The NOS examines methodological rigor across selection, comparability, and outcome ascertainment domains. Total scores vary from 1 to 9, and studies achieving ≥ 8 points were considered to be of high quality. Extracted data included study characteristics (first author, publication year, country, and study design), participant characteristics [source of the population, numbers of included participants, mean age, sex distribution, mean body mass index (BMI)], exposure assessment (methods for the diagnosis of OSAS and number of participants with OSAS), outcome validation (diagnostic criteria for sarcopenia and number of patients with sarcopenia), and covariates matched or adjusted when the association between OSAS and sarcopenia was analyzed.

### Statistical analyses

The association between OSAS and sarcopenia in adults was evaluated by combining odds ratios (ORs) and their corresponding 95% confidence intervals (CIs). When necessary, effect estimates and standard errors were derived from reported 95% CIs or *p*-values. All estimates were log-transformed before pooling to enhance normal distribution assumptions and stabilize variances (26). To evaluate variability across studies, we applied the Cochrane Q test and calculated the I^2^ statistic (28). I^2^ values below 25% were classified as low heterogeneity, 25–75% as moderate, and above 75% as high heterogeneity. Pooled effect estimates were calculated using a random-effects model to accommodate variability across studies (26). We performed leave-one-out sensitivity analyses, sequentially excluding individual studies to assess the robustness of the findings (29). In addition, a sensitivity analysis limiting to studies with high quality (NOS ≥ 8) was also performed. To identify possible sources of between-study variability, we performed predefined subgroup analyses stratified by study region (Asian vs. non-Asian countries), source of population (community population vs. clinical patients), mean age of the participants, proportions of men, diagnosis of OSAS (polysomnography or home sleep test vs. questionnaire or clinical records), diagnostic criteria for sarcopenia, analytic model (univariate or multivariate), and whether BMI was adjusted. In addition, univariate meta-regression analyses were performed to explore the potential influence of study-level characteristics on the association between OSAS and sarcopenia, including sample size, mean age, proportion of men, and NOS scores (26). To assess potential publication bias, we inspected funnel plots for asymmetry and conducted Egger’s regression analysis (30). A two-tailed *p*< 0.05 was considered statistically significant. All statistical analyses were performed using RevMan (version 5.3; Cochrane Collaboration, Oxford, United Kingodm) and Stata (version 17.0; StataCorp, College Station, TX, United States).

## Results

### Database search results

**FIGURE 1:**
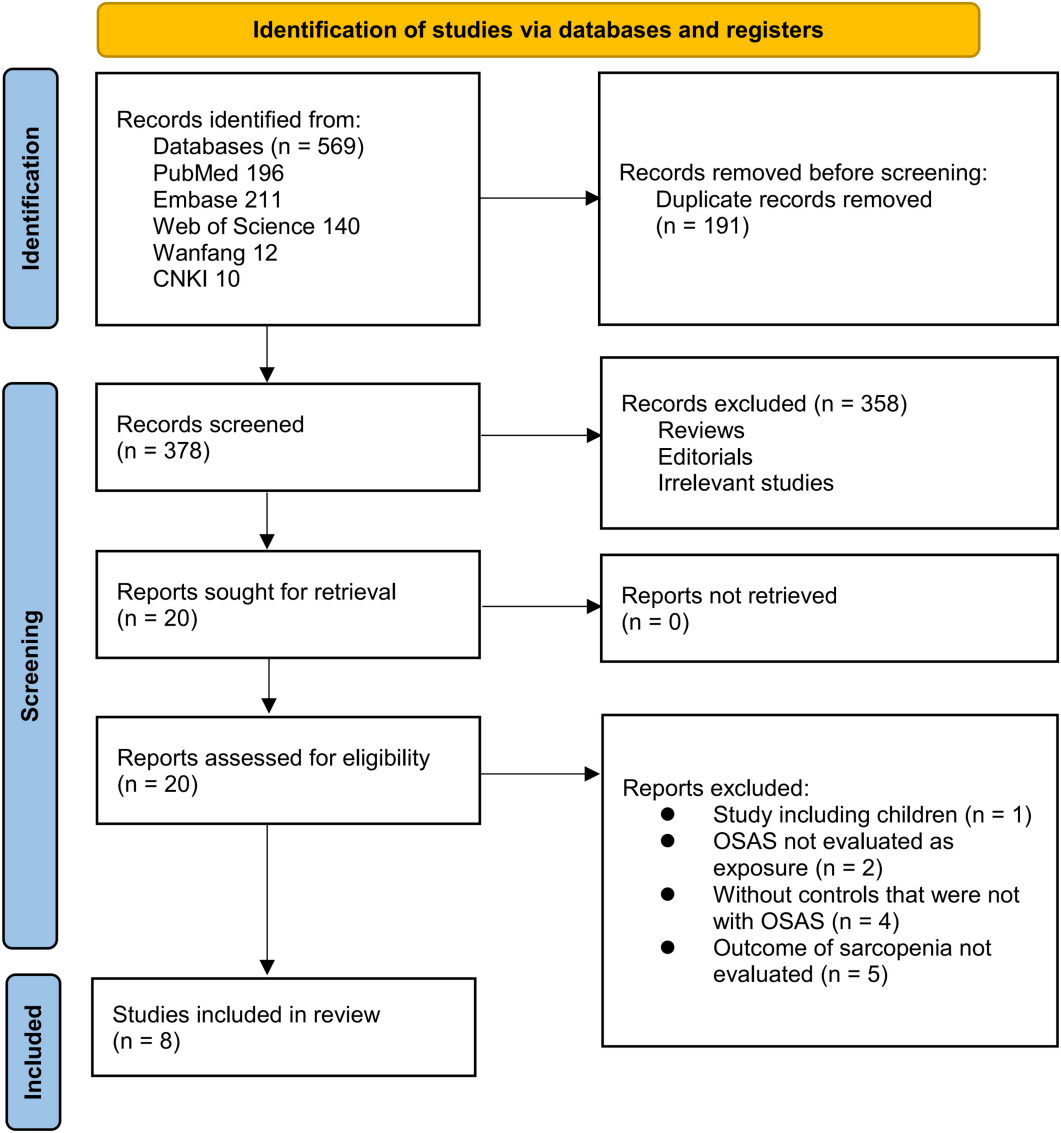
Flow diagram of the study selection process.

The study selection procedure is illustrated in Figure 1. A total of 569 records were retrieved from the five databases, and 191 duplicates were removed. Following screening of titles and abstracts, 358 records were excluded for failing to meet the predefined inclusion criteria. Twenty articles underwent full-text evaluation by two independent reviewers, after which 12 studies were excluded for the reasons detailed in Figure 1. Ultimately, eight studies met the eligibility criteria and were included in the quantitative meta-analysis (16–23).

### Overview of study characteristics

The characteristics of the included studies are summarized in Table 1. A total of eight cross-sectional studies (16–23) were included, published between 2019 and 2026, and conducted across Asia (China), South America (Brazil), North America (USA), and Europe (Spain), indicating broad geographic representation. The sample sizes varied substantially, ranging from 73 to 7,948 participants, with a combined population of 13,331 adults. The source populations differed across studies and included community-dwelling adults (18, 19, 21), geriatric inpatients (16), patients with T2D (20), CKD (17), bariatric surgery candidates (23), and specialty outpatient clinics (22). Mean age of participants ranged from 38.5 to 74.3 years, reflecting inclusion of both middle-aged and older adult populations. The proportion of men varied from 36.0 to 100%, and mean BMI ranged from 23.6 to 43.3 kg/m^2^, indicating inclusion of both non-obese and severely obese populations. OSAS was objectively diagnosed using polysomnography in four studies (16, 18, 20, 22) and home sleep study in one study (17), with AHI thresholds (AHI ≥ 5 or ≥ 15 events/hour). One study used the STOP-Bang questionnaire (19), one relied on self-reported symptoms (21), and one identified OSAS based on medical records (23). Accordingly, 3,572 (26.8%) subjects were with OSAS. Sarcopenia was diagnosed using established consensus criteria, including AWGS (16, 20), FNIH (18, 19, 21), EWGSOP (17), and EWGSOP2 (22, 23) definitions. Overall, 824 (6.2%) subjects were diagnosed as sarcopenia. Six studies (16–21) adjusted for key confounders such as age, sex, and BMI, and several additionally controlled for metabolic comorbidities, lifestyle factors, and laboratory parameters to a varying degree, although two studies (22, 23) reported results of univariate analysis only.

**TABLE 1:** Characteristics of the included studies.

Study	Country	Design	Source of population	No. of participants	Mean age (years)	Men (%)	Mean BMI (kg/m^2^)	Methods for the diagnosis of OSAS	No. of patients with OSAS	Methods for the diagnosis of sarcopenia	No. of patients with sarcopenia	Variables adjusted
Du et al. (16)	China	CS	Patients admitted to the Department of Geriatrics	320	74.3	49.7	NR	PSG (AHI ≥ 5)	39	AWGS criteria	110	Age, sex, alcohol consumption, BMI, T2D, osteoporosis, rheumatoid arthritis, and dementia
Piovezan et al. (18)	Brazil	CS	Community-dwelling adults	350	61.0	40.9	NR	PSG (AHI ≥ 15)	186	FNIH criteria	78	Age, sex, ethnic group, social class, education, marital status, smoking, alcohol, physical activity, number of comorbidities, depression score, medication use, and multiple serum biomarkers
Fernandes et al. (17)	Brazil	CS	Patients with CKD stages 3b-4	73	62.9	57.5	27.3	Home sleep study using a validated portable device (AHI ≥ 5)	49	EWGSOP criteria	9	Age, sex, eGFR, and BMI
Szlejf et al. (19)	Brazil	CS	Community-dwelling population	7948	59.5	46.3	27.7	STOP-Bang questionnaire	2011	FNIH criteria	128	Age, sex, race, education, leisure-time physical activity, current smoking, current alcohol intake, use of sedatives/hypnotics, and DM
Tian et al. (20)	China	CS	Patients with T2D	450	67.0	54.0	23.6	PSG (AHI ≥ 5)	29	AWGS criteria	42	Age, sex, smoking, BMI, ASMI, FPG, HbA1c, grip strength, and gait speed
Tao et al. (21)	USA	CS	Community-dwelling adults	3985	38.5	49.8	29	Self-reported symptoms from a questionnaire	1159	FNIH criteria	364	Age, sex, BMI, education, income, smoking, alcohol, caffeine, diet quality, physical activity, and comorbidities
Xue et al. (22)	China	CS	Outpatients from ENT & HN and orthopedic clinics	96	72.6	100.0	25.2	PSG (AHI ≥ 5)	50	EWGSOP2 criteria	47	None
Molero et al. (23)	Spain	CS	Candidates with severe obesity for primary bariatric surgery	109	64.1	36.0	43.3	Clinical diagnosed OSAS based on medical records	49	EWGSOP2 criteria	46	None

NR, not reported; CS, cross-sectional; BMI, body mass index; OSAS, obstructive sleep apnea syndrome; PSG, polysomnography; AHI, apnea–hypopnea index; AWGS, Asian Working Group for Sarcopenia; FNIH, Foundation for the National Institutes of Health; EWGSOP, European Working Group on Sarcopenia in Older People; EWGSOP2, European Working Group on Sarcopenia in Older People 2; CKD, chronic kidney disease; STOP-Bang, Snoring, Tiredness, Observed apnea, high blood Pressure, Body mass index, Age, Neck circumference, Gender questionnaire; T2D, type 2 diabetes; DM, diabetes mellitus; ASMI, appendicular skeletal muscle mass index; FPG, fasting plasma glucose; HbA1c, glycated hemoglobin; ENT, ear, nose, and throat; HN, head and neck.

### Study quality evaluation

Methodological quality was assessed using the NOS, and detailed results are presented in Table 2. The NOS scores ranged from 6 to 9, indicating overall moderate to high methodological quality. One study (20) achieved the maximum score of 9, reflecting strong case definition, appropriate selection of participants, adequate control for age and additional confounders, and reliable exposure ascertainment. Five studies (16–19, 21) scored 8, primarily due to minor limitations in representativeness, exposure assessment, or non-response reporting. One study scored 7 because of limited control for confounders (22). Another study (23) scored 6, mainly attributable to lack of adjustment for age and other confounders and less robust exposure ascertainment. Importantly, all included studies received full stars for adequate case definition, selection of controls, and consistent outcome ascertainment methods. Six studies appropriately controlled for age (16–21), which is a critical confounder in the association between OSAS and sarcopenia, and four studies also adjusted for BMI (16, 17, 20, 21) and other metabolic factors. Overall, the methodological quality of the included studies was considered moderate to high, supporting the credibility of the pooled estimates examining the association between OSAS and sarcopenia.

**TABLE 2:** Study quality evaluation via the Newcastle-Ottawa Scale.

Studies	Adequate definition of cases	Representa- tiveness of cases	Selection of controls	Definition of controls	Control for age	Control for other confounders	Exposure ascertainment	Same methods for events ascertainment	Non-response rates	Total
Du et al. (16)	1	1	1	1	1	1	1	1	0	8
Piovezan et al. (18)	1	0	1	1	1	1	1	1	1	8
Fernandes et al. (17)	1	1	1	1	1	1	1	1	0	8
Szlejf et al. (19)	1	1	1	1	1	1	0	1	1	8
Tian et al. (20)	1	1	1	1	1	1	1	1	1	9
Tao et al. (21)	1	1	1	1	1	1	0	1	1	8
Xue et al. (22)	1	1	1	1	0	0	1	1	1	7
Molero et al. (23)	1	1	1	1	0	0	0	1	1	6

### Meta-analysis results

**FIGURE 2:**
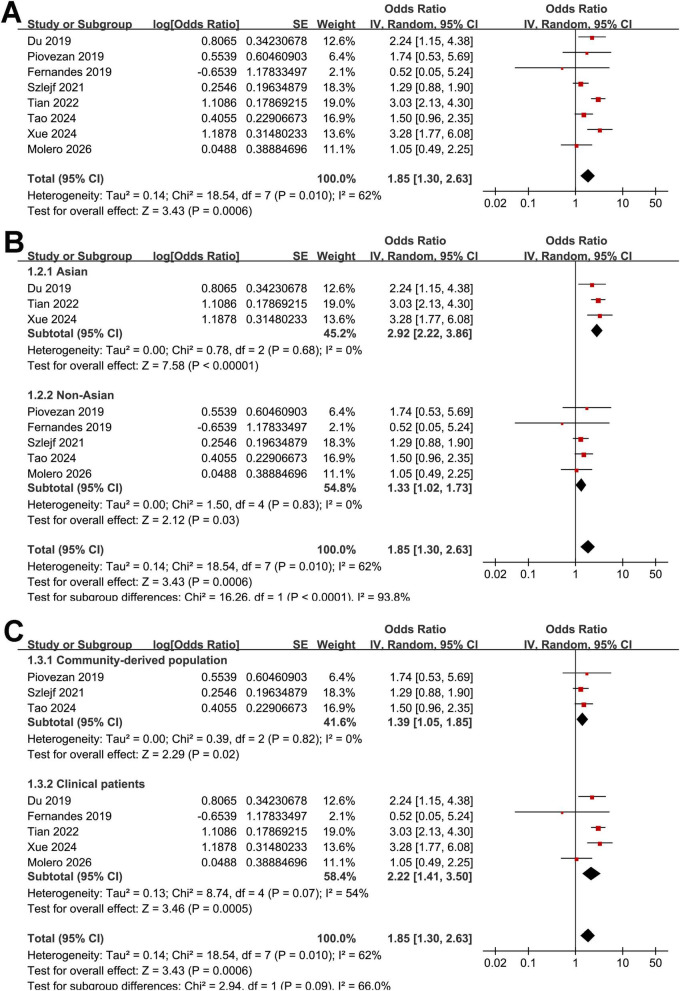
Forest plots showing the meta-analysis of the association between OSAS and sarcopenia in adults. **(A)** overall meta-analysis. **(B)** Subgroup analysis by study region. **(C)** Subgroup analysis by source of the population.

The pooled analysis of the eight studies (16–23) demonstrated that OSAS was significantly associated with sarcopenia in adult population (OR: 1.85, 95% CI: 1.30–2.63, *p* < 0.001; Figure 2A) with moderate heterogeneity (Cochrane Q test *p* < 0.01; *I*^2^ = 62%). Leave-one-out sensitivity analyses yielded consistent results, with pooled ORs ranging from 1.65 to 2.02 (all *p* < 0.05), indicating the robustness of the overall estimate. Notably, the sensitivity analysis limited to high quality studies with NOS ≥ 8 (16–21) showed consistent result (OR: 1.82, 95% CI: 1.23–2.70, *p* = 0.003; *I*^2^ = 62%).

**FIGURE 3:**
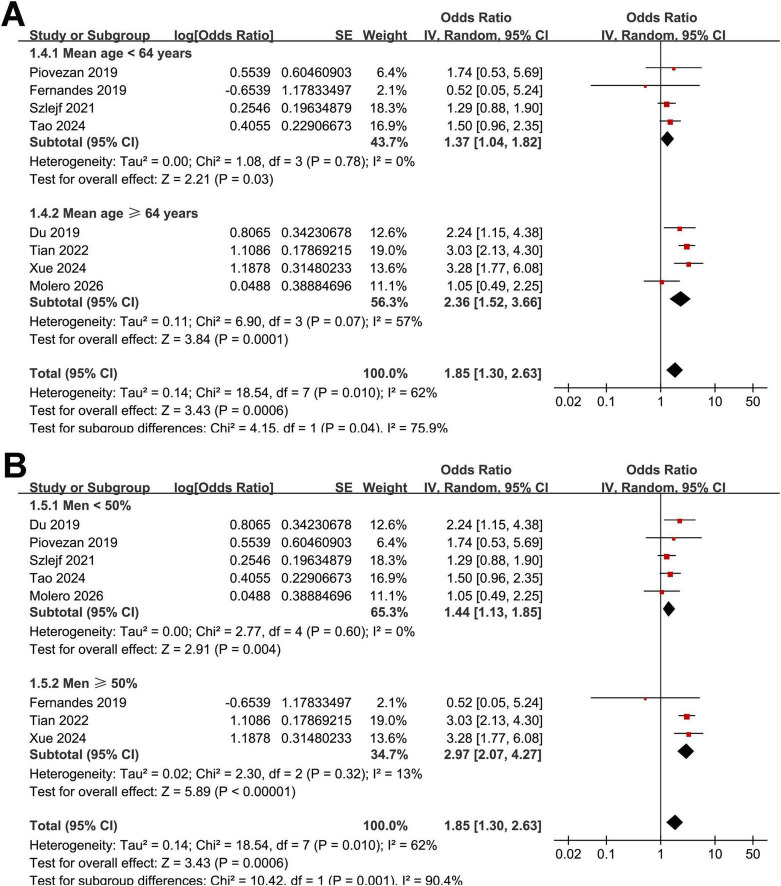
Forest plots for subgroup analyses of the association between OSAS and sarcopenia in adults. **(A)** Stratified by mean age of the populations. **(B)** Stratified by the proportions of men.

**FIGURE 4:**
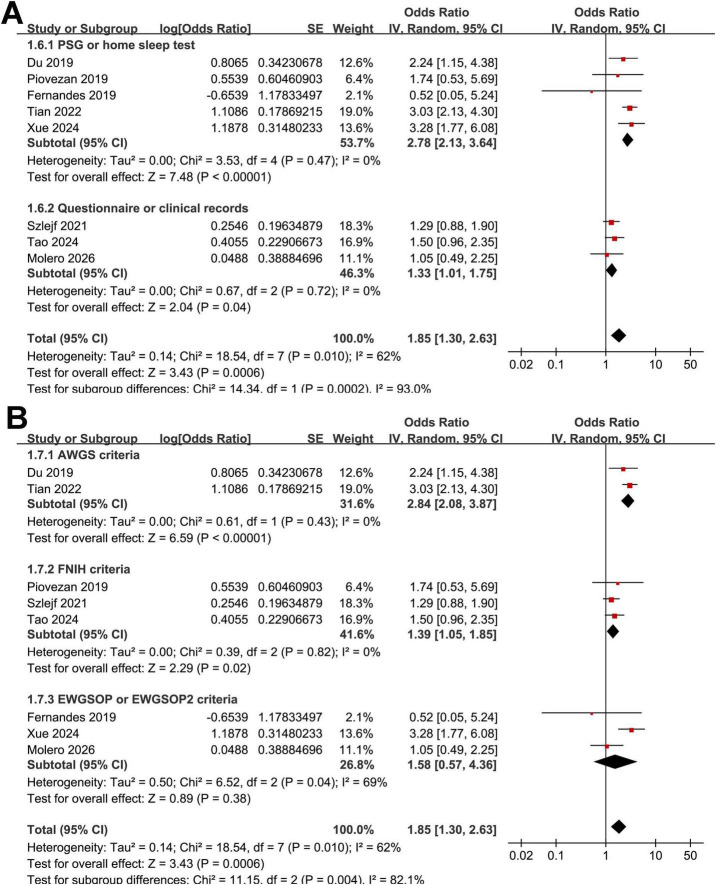
Forest plots for subgroup analyses of the association between OSAS and sarcopenia in adults. **(A)** Stratified by the diagnosis of OSAS. **(B)** Stratified by the diagnostic criteria for sarcopenia.

**FIGURE 5:**
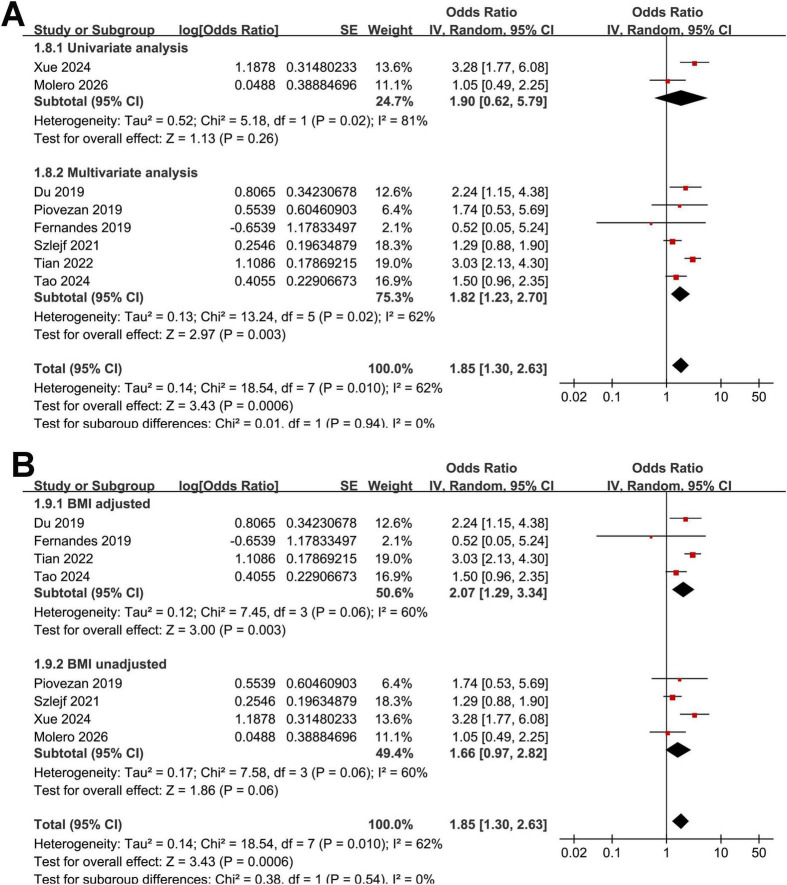
Forest plots for subgroup analyses of the association between OSAS and sarcopenia in adults. **(A)** stratified by the analytic models. **(B)** stratified according to whether BMI was adjusted.

Interestingly, the subgroup analysis showed a stronger association between OSAS and sarcopenia in studies from Asian countries as compared to those from non-Asian countries (OR: 2.92 vs. 1.33, *p* for subgroup difference < 0.001; Figure 2B). Heterogeneity was eliminated within each subgroup (*I*^2^ = 0%), while a significant between-subgroup difference was observed (*p* < 0.001), suggesting that geographic region may explain the overall heterogeneity. The results were not statistically different between studies with community-derived populations and those with clinical patients (OR: 1.39 vs. 2.22, *p* for subgroup difference = 0.09; Figure 2C). Further subgroup analysis suggested that the association between OSAS and sarcopenia might be stronger in older participants (mean age ≥ 64 years) as compared to younger ones (mean age < 64 years, OR: 2.36 vs. 1.37, *p* for subgroup difference = 0.04; Figure 3A). In addition, a stronger association was observed in studies with the proportion of men ≥ 50% as compared to those < 50% (OR: 2.97 vs. 1.44, *p* for subgroup difference = 0.01; Figure 3B). Moreover, a stronger association between OSAS and sarcopenia was also observed in studies with OSAS objectively diagnosed with polysomnography or home sleep test than those with OSAS diagnosed with questionnaire or clinical records (OR: 2.78 vs. 1.33, *p* for subgroup difference < 0.001; Figure 4A). Also, a stronger association was observed in studies with sarcopenia diagnosed with AWGS criteria as compared to FNIH or EWGSOP criteria (OR: 2.84 vs. 1.39 and 1.58, *p* for subgroup difference = 0.004; Figure 4B). Finally, the results were similar between studies with univariate or multivariate analyses (OR: 1.90 vs. 1.82, *p* for subgroup difference = 0.94; Figure 5A), and between studies with or without the adjustment of BMI (OR: 2.07 vs. 1.66, *p* for subgroup difference = 0.54; Figure 5B).

Meta-regression analyses did not identify any study-level characteristics as significant modifiers of the association, including sample size, mean age, proportion of men, and NOS score (Table 3, *p* all > 0.05), while the mean age of the participants and the proportion of men were found to largely explained heterogeneity (adjusted *R*^2^ = 49.3 and 48.2%, respectively). However, these findings should be interpreted cautiously given the limited number of included studies.

**TABLE 3:** Results of univariate meta-regression analysis.

Variables	OR for the association between OSAS and sarcopenia			
	Coefficient	95% CI	*p*-values	Adjusted R^2^
Sample size	−0.000076	−0.000194 to 0.000043	0.17	8.2%
Mean age (years)	0.018	−0.002 to 0.038	0.07	49.3%
Men (%)	0.015	−0.003 to 0.033	0.09	48.2%
NOS	0.19	−0.30 to 0.68	0.38	2.5%

OSAS, obstructive sleep apnea syndrome; OR, odds ratio; CI, confidence interval; NOS, Newcastle-Ottawa Scale.

### Publication bias

**FIGURE 6:**
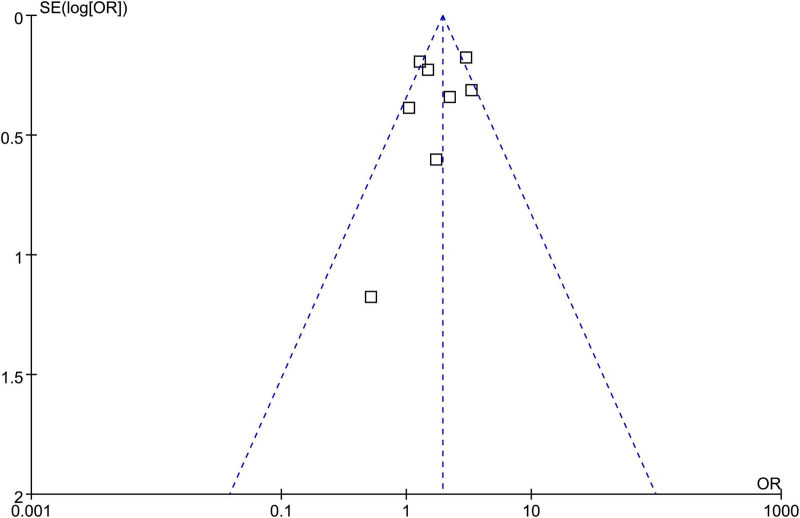
Funnel plots evaluating potential publication bias in the meta-analysis of the association between OSAS and sarcopenia in adults.

As shown in Figure 6, the funnel plots for the association between OSAS and sarcopenia appeared largely symmetrical. Consistent with this, Egger’s test did not indicate statistically significant asymmetry (*p* = 0.49). However, given the limited number of studies (*k* = 8), publication bias assessment should be interpreted cautiously.

## Discussion

This meta-analysis synthesizes current observational evidence and demonstrates that OSAS is associated with higher odds of sarcopenia in adults, with consistent findings across multiple sensitivity analyses. Although moderate heterogeneity was observed, the association remained stable after sequential exclusion of individual studies and when analyses were restricted to higher-quality studies, supporting the robustness of the pooled estimate. However, the cross-sectional design of all included studies precludes causal inference. Importantly, subgroup analyses suggested that the strength of association varied according to geographic region, age, diagnostic methods for OSAS, and criteria used to define sarcopenia, indicating that both biological and methodological factors may influence observed effect sizes.

Several pathophysiological mechanisms may plausibly explain the association between OSAS and sarcopenia. Chronic intermittent hypoxia, a hallmark of OSAS (31), promotes oxidative stress, systemic inflammation, and activation of catabolic signaling pathways, including nuclear factor-κB (32) and ubiquitin–proteasome systems (33), which may accelerate skeletal muscle protein degradation. Recurrent hypoxic episodes may also impair mitochondrial function (34) and reduce muscle oxidative capacity, contributing to muscle fatigue and atrophy (35). In addition, OSAS is associated with hormonal dysregulation, including alterations in growth hormone, testosterone, cortisol, and insulin-like growth factor-1 (36, 37), all of which are critical regulators of muscle mass and strength (38). Sleep fragmentation and excessive daytime sleepiness may further reduce physical activity levels, leading to disuse-related muscle loss (39). Beyond these direct effects, OSAS and sarcopenia share common risk factors, including aging, obesity, insulin resistance, and chronic low-grade inflammation, suggesting that shared metabolic and inflammatory pathways may partially mediate their co-occurrence (40).

The stronger association observed in Asian populations compared with non-Asian populations may reflect differences in body composition, genetic background, lifestyle patterns, and diagnostic thresholds for both OSAS and sarcopenia. Asian populations tend to have higher body fat percentages and greater visceral adiposity at lower BMI levels (41, 42), which may exacerbate metabolic dysfunction and inflammation, potentially amplifying the impact of OSAS on skeletal muscle. Differences in consensus definitions, particularly the use of AWGS criteria in several Asian studies (43), may also contribute to variation in effect estimates. The more pronounced association in older populations is biologically plausible, as aging is accompanied by reduced anabolic responsiveness, increased inflammatory burden, and impaired muscle regeneration (44), which may render older adults more vulnerable to the detrimental effects of intermittent hypoxia and sleep disruption. Furthermore, studies using objective sleep assessment methods (polysomnography or validated home sleep tests) demonstrated stronger associations than those relying on questionnaires or medical records (45), likely due to more accurate exposure classification and reduced misclassification bias. Similarly, variation in sarcopenia criteria may influence observed associations, as definitions emphasizing muscle strength and physical performance may better capture clinically meaningful muscle dysfunction than those based solely on muscle mass.

However, these findings should also be interpreted in light of potential misclassification bias arising from heterogeneous definitions of both exposure and outcome. While some studies used polysomnography or validated home sleep tests, others relied on questionnaires or medical records, which may have introduced exposure misclassification and attenuated or exaggerated the true association. Similarly, sarcopenia was defined using different consensus criteria (AWGS, EWGSOP/EWGSOP2, and FNIH), which differ in their emphasis on muscle mass, strength, and physical performance. These variations may influence case identification and limit the direct comparability and clinical interpretation of the pooled odds ratios across studies. In addition, residual confounding remains a concern. Although most included studies adjusted for age, sex, and BMI, other important factors such as physical activity, nutritional status, systemic inflammation, and severity of comorbid conditions were not consistently accounted for. Given that OSAS and sarcopenia share common risk factors, including obesity, metabolic dysfunction, and chronic inflammation, the observed association may partly reflect shared underlying pathways rather than a direct independent effect. Furthermore, the cross-sectional design of all included studies precludes determination of temporal relationships and raises the possibility of reverse causality. While OSAS-related intermittent hypoxia and sleep fragmentation may contribute to muscle catabolism, it is also plausible that reduced muscle mass and strength, particularly in respiratory and upper airway muscles, may exacerbate airway collapsibility and worsen sleep-disordered breathing. Therefore, a bidirectional relationship between OSAS and sarcopenia cannot be excluded.

This meta-analysis has several strengths. First, we conducted a comprehensive and up-to-date literature search across multiple international and Chinese databases, enhancing coverage and reducing the likelihood of missing relevant studies. Second, most included studies employed multivariate analyses and adjusted for key confounders such as age, sex, and BMI, increasing the reliability of the pooled estimates. Third, we performed multiple predefined subgroup and sensitivity analyses to explore potential sources of heterogeneity, and the association remained directionally consistent across these analyses. These methodological approaches enhance the robustness and credibility of the findings. However, several limitations should be acknowledged. First, moderate heterogeneity was observed across studies (*I*^2^ = 62%), which may reflect differences in study populations, comorbid conditions, and methodological approaches, although subgroup analyses partially accounted for this variability. Second, there was substantial heterogeneity in the diagnostic criteria and assessment methods for both OSAS and sarcopenia, which may have introduced misclassification bias and limited comparability across studies. Third, residual confounding cannot be excluded, as key factors such as physical activity, nutritional status, systemic inflammation, and disease severity were not consistently adjusted for, despite most studies controlling for age, sex, and BMI. Fourth, only eight studies were included, which may limit statistical power, particularly for subgroup analyses and publication bias assessment. Finally, due to limited reporting of OSAS severity metrics, such as the AHI, we were unable to perform dose–response analyses to evaluate whether increasing severity of OSAS is associated with a higher risk of sarcopenia. These limitations should be considered when interpreting the findings.

From a clinical perspective, our findings highlight the importance of heightened awareness of muscle health in patients with OSAS, particularly among older adults and those in Asian populations. Although the current evidence supports an association rather than causation, clinicians may consider screening for sarcopenia or muscle impairment in individuals with moderate-to-severe OSAS, especially when additional risk factors are present. Future research should prioritize well-designed prospective cohort studies to clarify temporal relationships and determine whether OSAS independently predicts incident sarcopenia. Interventional studies evaluating whether effective treatment of OSAS, such as continuous positive airway pressure therapy (46), can attenuate muscle decline would also provide valuable mechanistic and clinical insights. Standardization of diagnostic criteria and incorporation of objective measures of both sleep-disordered breathing and muscle function are essential to improve comparability across studies.

## Conclusion

In conclusion, this meta-analysis demonstrates that OSAS is associated with increased odds of sarcopenia in adults, with stronger associations observed in older individuals, Asian populations, and studies using objective sleep assessment. However, all included studies were cross-sectional, precluding assessment of temporal relationships, and there was heterogeneity in the diagnostic criteria for both OSAS and sarcopenia. Therefore, the findings should be interpreted cautiously and do not support causal inference. Further well-designed longitudinal studies with standardized definitions are needed to clarify the temporal relationship and underlying mechanisms.

## Data Availability

The original contributions presented in this study are included in the article/Supplementary material, further inquiries can be directed to the corresponding author.
