# Supplementary material for: The association between obstructive sleep apnea syndrome and sarcopenia: a meta-analysis
Source: Front Med (Lausanne). 2026 May 8;13:1815694. doi: 10.3389/fmed.2026.1815694 (PMC13193991; doi:10.3389/fmed.2026.1815694)
Supplement: Supplementary file 1 [file Table_1.docx]

**Detailed search strategy for each database**

**PubMed**

("Sleep Apnea, Obstructive"[Mesh] OR "Sleep Apnea Syndromes"[Mesh] OR "sleep disordered breathing"[tiab] OR "sleep-disordered breathing"[tiab] OR "sleep breathing disorder*"[tiab] OR "sleep apnea"[tiab] OR "sleep apnoea"[tiab] OR "obstructive sleep apnea"[tiab] OR "obstructive sleep apnoea"[tiab] OR "obstructive sleep apnea syndrome"[tiab] OR "obstructive sleep hypopnea syndrome"[tiab] OR OSAS[tiab] OR OSAHS[tiab] OR AHI[tiab]) AND ("Sarcopenia"[Mesh] OR sarcopenia[tiab] OR sarcopaenia[tiab] OR sarcopenic[tiab] OR presarcopenia[tiab] OR "Muscle Strength"[Mesh] OR "Muscle Weakness"[Mesh] OR "muscle wasting"[tiab] OR "muscle loss"[tiab] OR "muscle depletion"[tiab] OR "muscular atrophy"[tiab] OR "skeletal muscle mass"[tiab] OR "appendicular muscle"[tiab] OR "lean body mass"[tiab] OR "skeletal muscle index"[tiab] OR SMI[tiab] OR ASMI[tiab])

**Embase**

('obstructive sleep apnea'/exp OR 'sleep disordered breathing'/exp OR 'sleep apnea syndrome' OR 'sleep disordered breathing' OR 'sleep breathing disorder*' OR 'obstructive sleep apnea' OR 'obstructive sleep apnoea' OR OSAS OR OSAHS OR AHI) AND ('sarcopenia'/exp OR 'muscle weakness'/exp OR 'muscle mass'/exp OR sarcopenia OR sarcopenic OR presarcopenia OR 'muscle wasting' OR 'muscle loss' OR 'skeletal muscle mass' OR 'lean body mass' OR 'skeletal muscle index' OR SMI OR ASMI)

**Web of Science**

TS=(("sleep disordered breathing" OR "sleep-disordered breathing" OR "sleep breathing disorder*" OR "sleep apnea" OR "sleep apnoea" OR "obstructive sleep apnea" OR "obstructive sleep apnoea" OR "obstructive sleep apnea syndrome" OR "obstructive sleep hypopnea syndrome" OR OSAS OR OSAHS OR AHI) AND ("sarcopenia" OR "sarcopaenia" OR "sarcopenic" OR "presarcopenia" OR "muscle wasting" OR "muscle loss" OR "muscle depletion" OR "muscular atrophy" OR "skeletal muscle mass" OR "lean body mass" OR "skeletal muscle index" OR SMI OR ASMI))

Wanfang

("阻塞性睡眠呼吸暂停" OR "阻塞性睡眠呼吸暂停综合征" OR "睡眠呼吸暂停" OR "睡眠呼吸障碍" OR OSAS OR OSAHS) AND ("肌少症" OR "肌肉减少症" OR "肌肉萎缩" OR "骨骼肌减少" OR "骨骼肌指数" OR "肌肉质量")

China National Knowledge Infrastructure (CNKI)

("阻塞性睡眠呼吸暂停" OR "阻塞性睡眠呼吸暂停综合征" OR "睡眠呼吸暂停" OR "睡眠呼吸障碍" OR OSAS OR OSAHS) AND ("肌少症" OR "肌肉减少症" OR "肌肉萎缩" OR "肌肉减少" OR "骨骼肌减少" OR "骨骼肌指数" OR "肌肉质量")
